# The impact of positive and negative testimony on children’s attitudes toward others

**DOI:** 10.1371/journal.pone.0261075

**Published:** 2021-12-22

**Authors:** Asami Shinohara, Yasuhiro Kanakogi, Yuko Okumura, Tessei Kobayashi

**Affiliations:** 1 NTT Communication Science Laboratories, Kyoto, Japan; 2 Graduate School of Human Sciences, Osaka University, Osaka, Japan; Bucharest University of Economic Studies, ROMANIA

## Abstract

Children can identify who is benevolent or malevolent not only through first-hand experiences and observations but also from the testimony of others. In this study, we investigated whether 5- and 7-year-olds (*N* = 128) would form their attitudes toward others after hearing testimony about that person’s past moral behavior and whether the valence of testimony would differently influence the children. In the positive condition, half of the participants gained information about three puppets: puppet A’s prosocial behavior by their own first-hand observation, testimony about puppet B’s past prosocial behavior, and testimony about puppet C’s past neutral behavior. In the negative condition, the other half also learned information about the three puppets: puppet A’s antisocial behavior by their own first-hand observation, testimony about puppet B’s past antisocial behavior, and testimony about puppet C’s past neutral behavior. Then they engaged in tasks that measured their behavioral attitudes toward the puppets and evaluated the goodness of each puppet to assess their attitudes at a cognitive level. Our results concluded that the children form their behavioral attitudes toward others based on testimony starting at the age of 7, and attitude formation at the cognitive level based on testimony is seen at age 5. Negative testimony, rather than positive testimony, influences the children’s attitudes toward others. In addition, the 7-year-olds’ use of testimony differs depending whether they are the allocators or the receivers of rewards. Our findings deepen understanding of how children rely on the verbal information around themselves when they navigate interactions with others.

## Introduction

Children learn about, evaluate, and form attitudes toward individuals with whom they are involved in their social lives. Through direct observation, children identify who is benevolent and who is malevolent. For instance, they categorize a person who showed prosocial behavior as nice and another who exhibited antisocial behavior as mean [1]. In addition, they are more likely to share resources with a benevolent person than a malevolent one [2, 3]. Unfortunately, it is impossible to observe the behavior of every person and learn about his/her personal traits considering the size of human societies [e.g., 4]. Relying on verbal information from others (i.e., testimony) can be beneficial and effective for identifying good people and avoiding the bad [5]. More importantly, forming attitudes based on such testimony is crucial to live smoothly in a group life [6].

Indeed, testimony about individuals’ personal traits is ubiquitous in human society; two-thirds of adult conversations are concerned with the personalities or experiences of others, including the speakers themselves or third-parties [7]. Adults display positive or negative attitudes toward a person, for example, by cooperating with or refusing cooperation, based on descriptions of his/her past cooperative or uncooperative behavior [8, 9]. Thus, individuals exploit such testimony when they need to develop attitudes toward others.

Not only adults but also children may be exposed to the testimony about others in their social lives. A previous study found that even toddlers talk about personal traits, such as nice or mean, for the characters in story books [10], suggesting that humans tend to verbally refer to the personal traits of others from an early age. Engelmann and his colleagues [11] experimentally showed that 5-year-olds provided verbal information about social agents (i.e., puppets) to a peer and described an agent’s benevolent or malevolent character based on past prosocial or antisocial behavior. Such a tendency in children suggests that even in their social lives, they are exposed to testimony about the actions of their peers, which makes it possible for them to form their own positive or negative attitudes toward others.

Do children actually form positive or negative attitudes toward those who are verbally described as benevolent or malevolent? One previous study [12] investigated children’s use of testimony about a social agent (i.e., a puppet). They examined whether 5- and 7-year-olds were more inclined to trust information about the moral behavior of others from first-hand observations as opposed to testimony when choosing a partner who was likely to cooperate with them. The children either obtained one positive piece of information (i.e., past prosocial behavior) about others through first-hand observation and one negative piece of information (i.e., past antisocial behavior) through testimony, or they obtained positive information through testimony and negative information through first-hand observation. Their results showed that the 5-year-olds trusted testimony about agents’ negative character more than the directly observed positive behaviors of the agents; the 7-year-olds trusted their own observations more than testimony regardless of the valence of information. These findings indicate that 5-year-olds are more likely to be influenced by negative information than positive information, whereas 7-year-olds demonstrate an understanding of how testimony about social agents (called gossip in their paper) should be treated; it can sometimes be treated with skepticism when such information contradicts their own direct observations [13]. However, in their study, the children only had to choose between one of two pieces of information with opposite content (a positive or negative trait of others) from different sources (one gained by their first-hand observation and another from testimony) when they chose a cooperative partner. From such methodology, it cannot be concluded whether children form their attitudes toward others based on each valence of testimony (positive/negative). In addition, although other studies [14, 15] demonstrated that children older than 6.5 expressed negative attitudes toward one member of a group based on negative testimony, it is unclear whether they rely on positive testimony when they form attitudes toward social agents, and if so, which valence is more influential on their attitudes.

Investigating how the valence of testimony influences children’s attitudes is necessary to understand how they selectively establish reciprocal relationships with the people around them, since there are various kinds of information about other’s moral traits and children need to decide how to interact with such others [16]. Actually, children are far more inclined to focus on and use negative information than positive information, a process called negativity bias [17]. Some evidence suggests that children exhibit negativity bias when they form their attitudes toward others [18–20]. For instance, children are more biased by negative non-verbal cues than positive ones when they rate the niceness of peers [20]. These facts suggest that children’s attitudes toward others are much more biased by negative testimony than positive testimony. On the other hand, according to positivity bias theory which exists in childhood [21], children attend to or more selectively process positive information than negative information when they judge an individual’s personal traits [22]. If positivity bias theory is also true in terms of the effect of verbal information, children would be more influenced by testimony about positive traits than negative ones.

In our study, we investigated whether children form attitudes toward a social agent (i.e., a puppet) after hearing positive or negative testimony about it. We assessed children’s attitudes toward others with both behavioral and cognitive aspects. To examine their attitudes expressed as their actual behavior, we measured how they allocated and anticipated rewards to delve into the underlying mechanism to establish reciprocal relationships [23]. When children try to build reciprocal relationships with others, they may need to consider to whom they should give benefits based on the moral traits of others (reward-allocation) [16] as well as to identify who is willing to confer benefits to themselves (reward-anticipation) [24] because such selective attitudes eventually nurture good reciprocal relationships for the children themselves in a long term. Thus, we employed reward-allocation and anticipation tasks to measure the children’s behavioral attitudes. In addition, to measure how they perceived others (i.e., their attitudes of the cognitive aspect), they evaluated the goodness of agents (explicit evaluation) [15]. We targeted 5- and 7-year-olds as our research sample. Since 5-year-olds are the youngest age that has been identified to have provided testimony about people [11], they have also probably learned to distinguish between benevolent and malevolent people based on testimony in their daily lives. Since previous studies [12, 15] described the developmental differences of the use of testimony between the ages of 5 and 7, a developmental difference would be seen to the extent that children are influenced by testimony. In addition, positivity and negativity biases are also found in children from 5 to 7 [18, 22], suggesting that examining this age range is also suitable to determine which bias would be true concerning the effect of verbal information on children.

Our main concern was how the positive and negative testimony would influence the children’s attitudes toward others. First, we tested whether children’s attitudes would differ depending on the content of testimony by comparing their attitudes toward a person whose past positive/negative behavior was described in testimony and whose past neutral behavior was described in testimony. Second, if we confirmed that testimony influenced the children’s attitudes, then we compared the impact of the positive/negative testimony with the positive/negative information based on first-hand observation. Third, we examined which valence of testimony (i.e., positive or negative) more greatly influenced their attitudes. In addition, we analyzed how differently the testimony would influence the attitudes of 5- and 7-year olds to find any developmental changes between the two age groups.

## Method and materials

### Participants

Sixty-four 5-year-olds (*M* = 5.30 years, *SD* = 0.17; 33 boys) and 64 7-year-olds (*M* = 7.40 years, *SD* = 0.28; 31 boys) participated in this study, most of whom came from middle-class Japanese families living in a small-sized city. Since a prior power analysis with a medium effect size based on previous studies [12, 15], *alpha* = .05, and *power* = .80, revealed that at least 31 participants are necessary in each condition for each age group, we collected data from 128 participants in total. The children were randomly assigned to either the positive or negative condition. We tested an additional six 5-year-olds and one 7-year-old but excluded them from the final sample because of their reluctance to join (three 5-year-olds), experimental error (one 5-year-old and one 7-year-old), and an inability to pass the memory check (two 5-year-olds). All the participants were recruited from a database of parents who agreed to join children’s studies. Before the experiment, all the parents provided written informed consent. This study was approved by an ethical committee of NTT Communication Science Laboratories (Study Number H26-002).

### Materials

The experimental room contained a table at which participants sat during the experiment. All the videos were played on an iPad Pro 12.9-inch tablet. Six boxes (10×10×3.5 cm) were used for the reward-allocation and reward-anticipation tasks. We used five puppets. Three focal puppets played the following roles: puppet A displayed moral behavior (prosocial or antisocial), puppet B’s moral behavior (prosocial or antisocial) was conveyed by testimony, and puppet C’s neutral behavior was conveyed by testimony. The puppets wore different colored shirts for easy discrimination (red, green, or yellow). One of the other two was the target of a focal puppet’s prosocial or antisocial behavior (puppet T), and another provided testimony (i.e., informant puppet; puppet I).

### Video stimuli

The children consecutively watched three different video clips: first-hand observation, testimony with valence (positive or negative testimony), and neutral testimony. The children in the positive condition watched positive first-hand observation and positive testimony videos, while the children in the negative condition watched negative first-hand observation and negative testimony videos. In both conditions, the children watched additional neutral testimony video. Specifically, the children gained information about three agents: positive or negative information about puppet A from first-hand observations, positive or negative information about puppet B from testimony, and neutral information about puppet C from testimony. We counterbalanced the presentation order of the video content and the role of each focal puppet across the children. We created two types of stimuli for each video, and the children watched one of two types for each video (S1 Table).

#### Positive condition; first-hand observation

First, puppet A stood on the right of the stage and the help-receiving puppet (puppet T) was on its left. Puppet T was playing with a ball/writing with a pencil, but it dropped the ball/pencil. After puppet A picked up the dropped object, puppet T said, “Thank you.”

#### Positive condition; positive testimony

Puppet I (i.e., the informant puppet) was in the center of the screen. Then puppet B passed in front of puppet I from left to right. After puppet B left the screen, puppet I provided positive testimony: “That kid helped his friend by picking up her ball/pencil a little while ago.” The children who watched the first-hand observation video relevant to the ball heard testimony about the puppet who picked up the pencil, and those who watched the first-hand observation video relevant to the pencil heard testimony about the puppet who picked up the ball. Because we wanted the information to be equivalent between the first-hand observations and the positive testimony, we employed verbal information that described the situation of the first-hand observation video.

#### Negative condition; first-hand observation

These videos started with puppet A on the right-hand side of the stage and the victim puppet (puppet T) on the left. In one video, puppet T placed the last block in its castle and announced to puppet A: “I’m finished.” Then puppet A destroyed the castle, and puppet T expressed sadness. In another video, puppet T was playing with a red ball while puppet A watched. As puppet A started to take puppet T’s red ball, puppet T protested, “Don’t.” After puppet A finally got the ball, puppet T expressed sadness.

#### Negative condition; negative testimony

This video was basically identical to the video of positive testimony, except for the content of testimony. Puppet I provided the following negative testimony about puppet B: “That kid took his friend’s ball a little while ago” or “That kid knocked down his friend’s castle of blocks a little while ago.” The children who watched the first-hand observation video relevant to building the castle heard testimony about the puppet taking the ball, and those watching the first-hand observation video relevant to the ball heard testimony about the puppet destroying the castle.

#### Neutral testimony

This video was basically identical to the testimony with the valence video, except for the content of testimony and its target. Puppet I provided neutral testimony about puppet C: “That kid was playing on a swing a little while ago” or “That kid was taking a walk a little while ago.

### Procedure

The study had a 2 (condition: positive or negative; between-subject) × 2 (age: 5- or 7-years-old; between-subject) × 3 (puppet: first-hand observation, testimony with valence, and neutral testimony; within-subject) design. The experiment was conducted by a female experimenter. All the children were first taken to the experimental room and told that they were going to play with three friends (i.e., puppets) and boxes filled with stickers. The experiment consisted of *puppet introduction*, *reward tasks*, and *explicit evaluation*.

#### Puppet introduction

The children watched the videos explained above twice. Then the experimenter introduced the actual puppets (puppet A, B and C) to the children and asked what they had done in the video or what the informant puppet (puppet I) had said about them. If their answers were incorrect, she showed the video again and repeated the questions about the video content.

#### Reward tasks

After the *puppet introduction*, the children completed the *reward-allocation* and *reward-anticipation* tasks that measured their attitudes toward the puppets. The order of the tasks was counterbalanced across the children. The experimenter first showed the children three boxes with red ribbons and three boxes with blue ribbons that were behind each puppet and explained that they were going to do some tasks with the puppets.

In the reward-allocation task, we differentiated the gift quantity by having the experimenter show the participants three boxes that contained the following items: 40 stickers (high reward), 10 stickers (medium reward), and one sticker (low reward). Our pilot study with 5-year-olds (*n* = 12) clearly showed that all the children understood that the high reward was the most valuable and that the low reward was the least valuable. They were asked to decide which puppets deserved the high, medium, and low rewards. The children set the rewards in front of the puppet chosen to receive each box. While they were engaged in this task, the experimenter looked away to avoid biasing their choices.

In the reward-anticipation task, the experimenter showed the children three identical opaque boxes whose contents were hidden from the children. Each box, which contained a gift, was set in front of each puppet. The experimenter explained that each box had a gift for the children from each puppet and asked them to point at the boxes they most wanted (high- reward-anticipation), their second choice (medium-reward-anticipation), and their least desired choice (low-reward-anticipation) from the focal puppet. The children then received their first choice. All the boxes contained two stickers. In this task, the experimenter did not allow the children to look into or touch the boxes to prevent them from guessing the content. The order of introducing the boxes from the puppets was counterbalanced.

#### Explicit evaluation

We asked the children to rate the goodness of each puppet on a 5-point Likert scale that contained three stars (2: *very good*), one star (1: *good*), a square (0: *neither*), one x-mark (-1: *bad*), and three x-marks (-2: *very bad*).

### Data analysis plan

#### Reward-allocation task

If the children in the positive condition allocated rewards based on the content of testimony, they should have given the low reward to puppet C (in the neutral testimony video). If the children in the negative condition allocated rewards based on the content of testimony, they should have given the high reward to puppet C. Therefore, we compared the number of children who allocated the low reward to puppets A, B, and C in the positive condition. In negative condition, we compared the number of children who allocated the high reward to puppets A, B, and C in the negative condition.

In addition, to examine whether they displayed a positivity or negativity bias when allocating rewards, we compared the number of children who gave the low reward to puppet C out of all the participants in the positive condition (i.e., the children who allocated rewards based on positive testimony) and those who gave the high reward to puppet C out of all the participants in the negative condition (i.e., the children who allocated rewards based on negative testimony).

#### Reward-anticipation task

If the children in the positive condition anticipated rewards based on the content of testimony, they should have chosen a reward from puppet C as their third choice. If the children in the negative condition anticipated rewards based on the content of testimony, they should have chosen a reward from puppet C as their first choice. Therefore, we compared the number of children who chose a reward from puppets A, B, and C as their third choice (low reward anticipation) in the positive condition. In the negative condition, we compared the number of children who chose a reward from puppets A, B, and C as their first choice (high reward anticipation) in the negative condition.

In addition, to examine whether they displayed a positivity or negativity bias when anticipating rewards, we compared the number of children who chose a reward from puppet C as their third choice out of all the participants in the positive condition (i.e., the children who anticipated rewards based on positive testimony) and those who chose a reward from puppet C as their first choice out of all the participants in the negative condition (i.e., the children who anticipated rewards based on negative testimony).

## Results

### Reward-allocation

**Fig 1:**
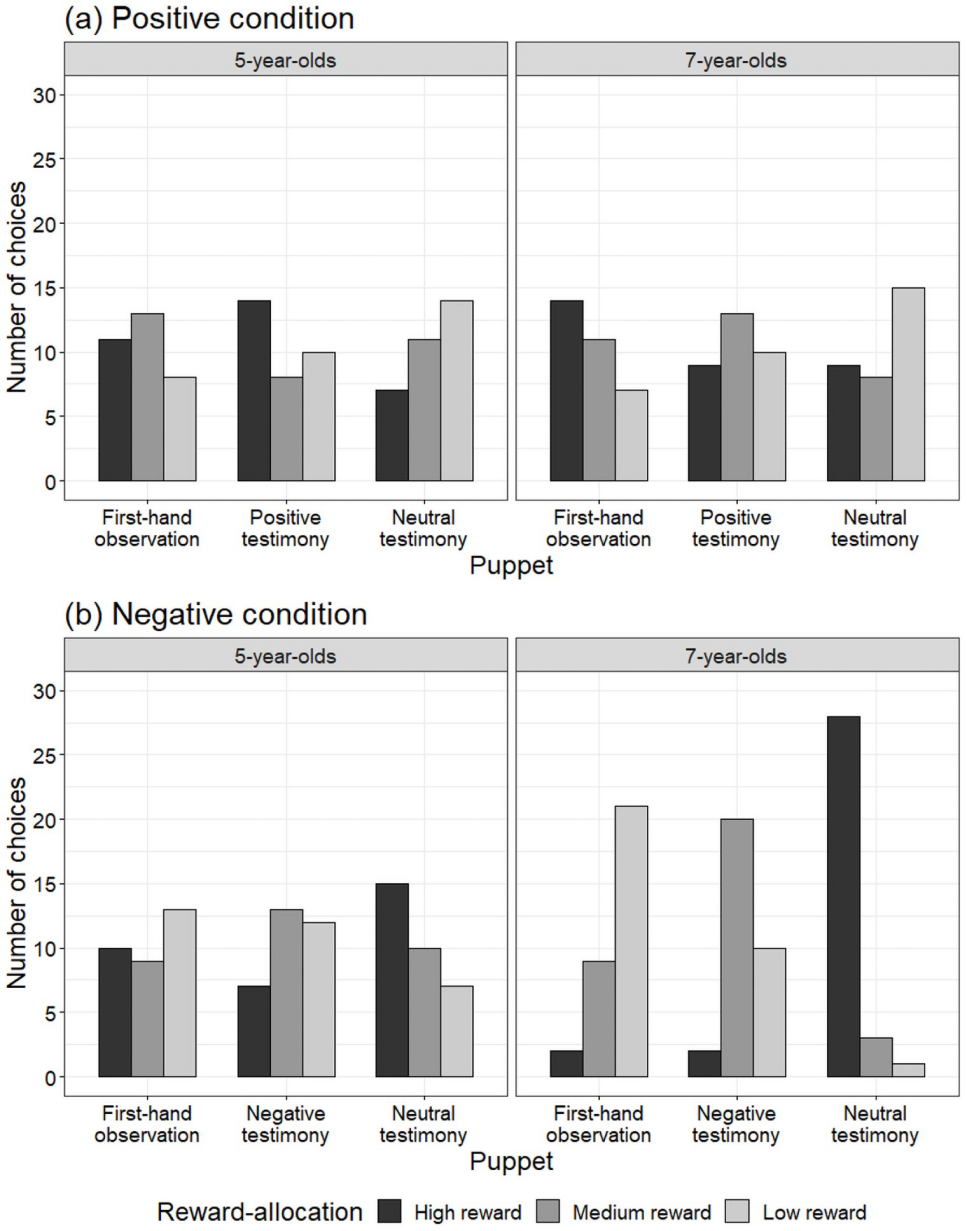
Results of reward-allocation task in (a) positive and (b) negative conditions. Each bar indicates number of children who chose each puppet at each decision point.

Fig 1(a) represents the results of the reward-allocation task in the positive condition. To determine whether a developmental difference existed in the pattern of the low-reward-allocation between the two age groups, we first conducted a 2 (age: 5- or 7-years-old) × 3 (puppet: first-hand observation, positive testimony, and neutral testimony) chi-square test on the number of children at the low-reward-allocation in the positive condition. Our analysis did not find a significantly different pattern of low-reward-allocation among them, χ^2^(2) = 0.10, *p* = .95. Therefore, subsequent analysis was collapsed across ages. To reveal whether the children gave the low reward to puppet C whose neutral behavior was conveyed by testimony, we conducted a one-way likelihood ratio chi-square on the number of children in the low-reward-allocation. Again, we found no differences among the number of children who allocated the low reward to the puppets in the first-hand observation (*n* = 15), in the positive testimony video (*n* = 20), or in the neutral testimony video (*n* = 29), χ^2^(2) = 4.72, *p* = .09.

Fig 1(b) represents the results of the reward-allocation task in the negative condition. To determine whether a developmental difference existed in the pattern of the high-reward-allocation between two age groups, we first conducted a 2 (age: 5- or 7-years-old) × 3 (puppet: first-hand observation, positive testimony, and neutral testimony) chi-square test on the number of children in the high-reward-allocation in the negative condition. The analysis found a significantly different pattern of high-reward-allocation among them, χ^2^(2) = 12.04, *p* < .01. To reveal whether the children allocated the high reward to puppet C (in the neutral testimony video), we separately conducted a one-way likelihood ratio chi-square on the number of 5- and 7-year-olds in the high-reward-allocation. The analysis of the 5-year-olds did not find any differences among the number of children who allocated the high reward to the puppets in the first-hand observation video (*n* = 10), in the negative testimony video (*n* = 7), or in the neutral testimony video (*n* = 15), χ^2^(2) = 3.06, *p* = .22. On the other hand, the analysis of the 7-year-olds did reveal significant differences among the number of children, χ^2^(2) = 42.25, *p* < .001. Most children gave the high reward to puppet C (in the neutral testimony video; *n* = 28, *p* < .001). Only 2 children gave the high reward to puppet A (in the first-hand observation video), and 2 gave it to puppet B (in the negative testimony video). Our next question addressed whether the impact of the negative testimony would be higher or lower than the negative information gained by first-hand observation for those who used the testimony to form their attitudes (i.e., those who allocated the high reward to puppet C in the neutral testimony video, *n* = 28). We conducted a one-way likelihood ratio chi-square on the number of 7-year-olds who gave the medium reward to either puppet in the first-hand observation or in the negative testimony video (Fig 3). The analysis found that the number of children who gave the medium reward to puppet B (in the negative testimony video: *n* = 20) was significantly higher than those who gave such reward to puppet A (in the first-hand observation video: *n* = 8), χ^2^(1) = 5.14, *p* = .023. The negative information gained from first-hand observation had more influence on the children’s attitudes.

Finally, we conducted a Fisher’s exact test to reveal whether the children displayed a positivity or negativity bias when they allocated rewards based on testimony. The number of 7-year-olds whose reward-allocations were influenced by negative testimony (88%) was significantly greater than the 7-year-olds whose reward-allocations were influenced by positive testimony (47%), *p* = .001. There were no significant differences between the number of 5-year-olds influenced by positive testimony (44%) and negative testimony (47%), *p* = 1.

### Reward-anticipation

**Fig 2:**
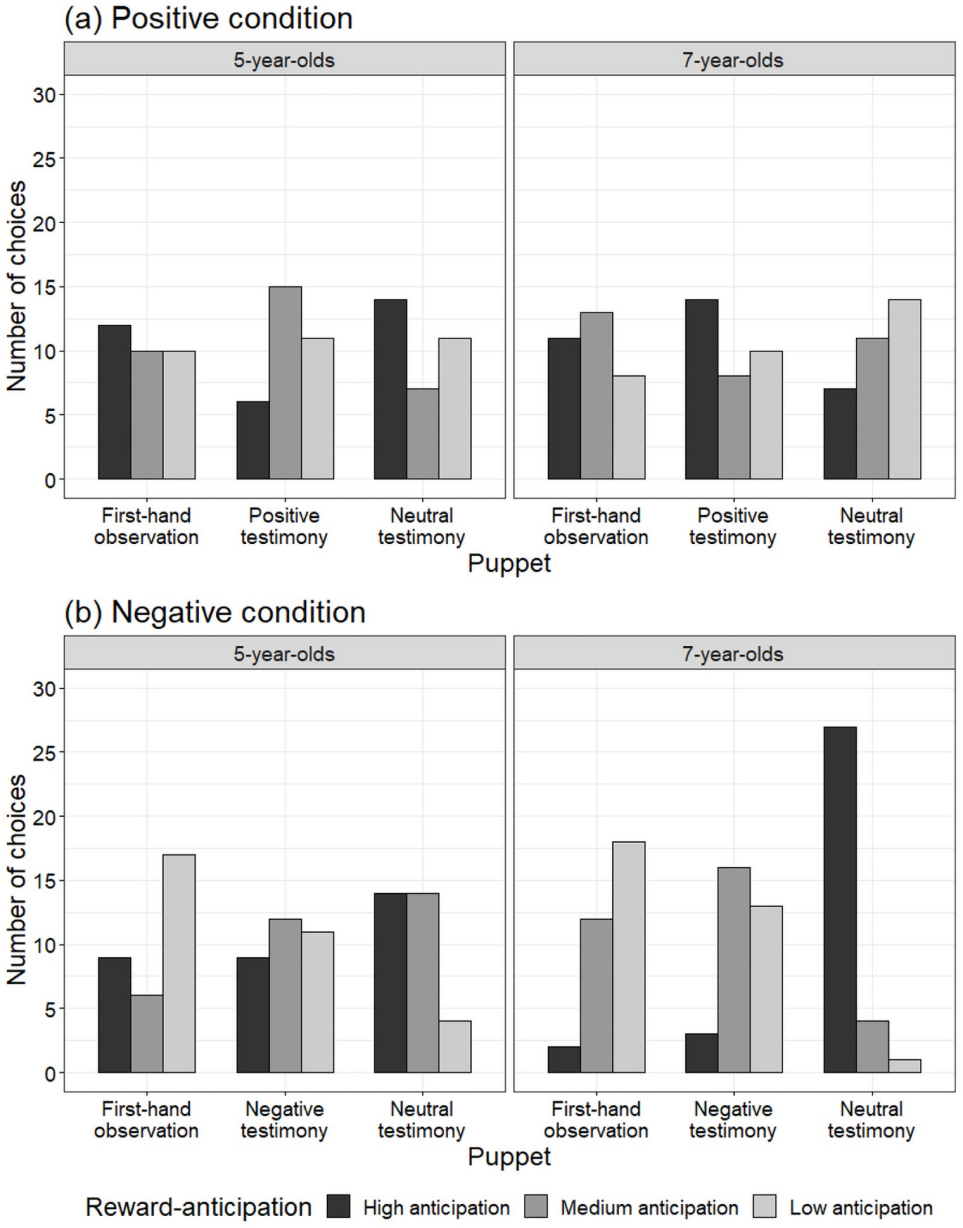
Results of reward-anticipation task in (a) positive and (b) negative conditions. Each bar indicates number of children who chose each puppet at each decision point.

Fig 2(a) represents the results of the reward-anticipation task in the positive condition. A 2 (age: 5- or 7-years-old) × 3 (puppet: first-hand observation, positive testimony, and neutral testimony) chi-square test on the number of children in the low-reward-anticipation in the positive condition found no significant difference in the pattern of low-reward-anticipation between the two age groups, χ^2^(2) = 2.29, *p* = .32. Next we conducted a one-way likelihood ratio chi-square on the number of children in the low-reward-anticipation to reveal whether they anticipated the low reward from puppet C whose neutral behavior was conveyed by testimony. The analysis found no significant differences among the number of children who anticipated the low reward from the puppets in the first-hand observation video (*n* = 17), in the positive testimony video (*n* = 19), or in the neutral testimony video (*n* = 28), χ^2^(2) = 3.22, *p* = .20.

**Fig 3:**
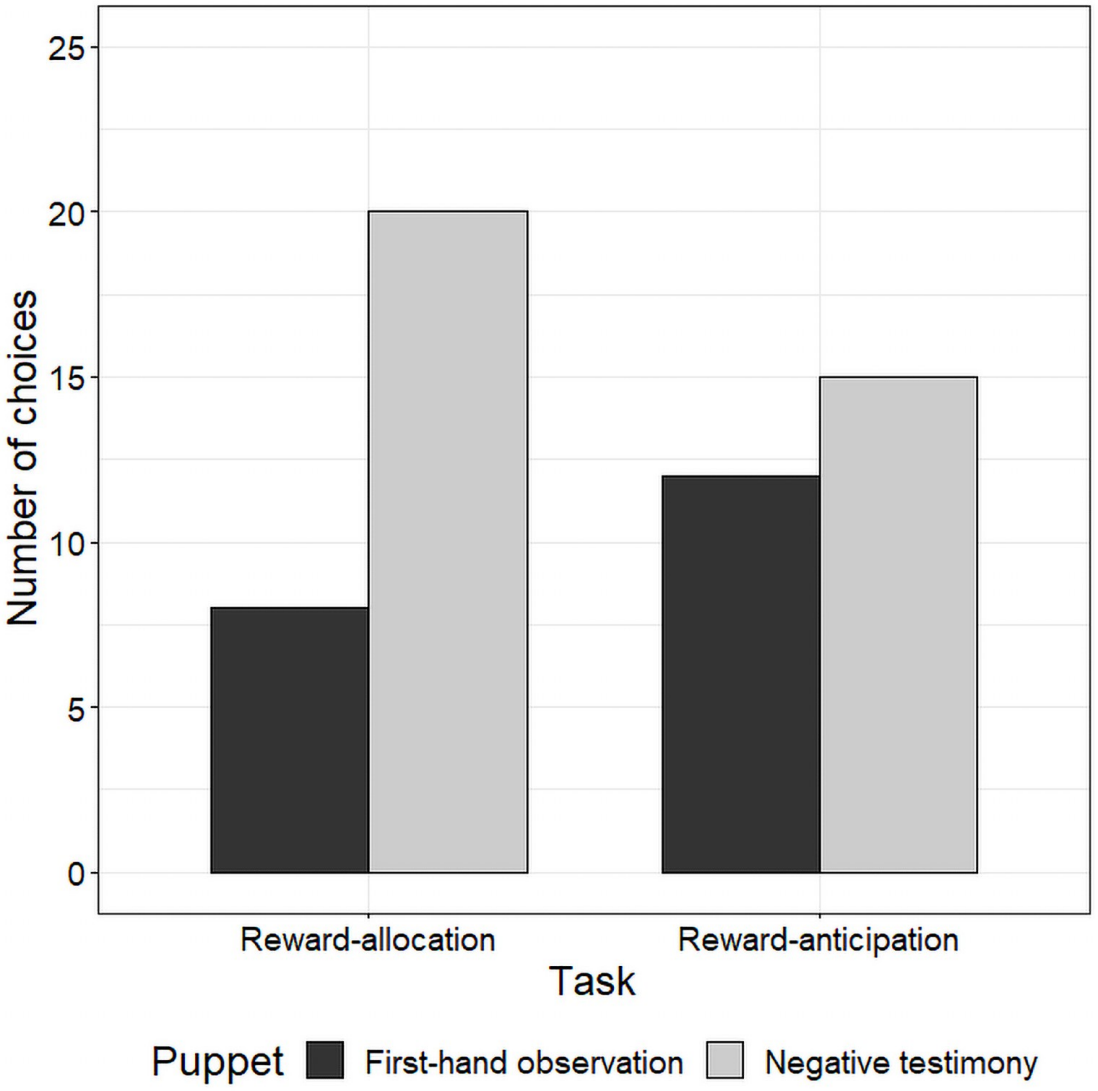
Number of children who gave the medium reward either to first-hand observation or negative testimony puppets in reward-allocation task and number of children who anticipated the medium reward either from first-hand observation or negative testimony puppets in reward-anticipation task. Each bar indicates number of children who chose each puppet at each decision point.

Fig 2(b) represents the results of the reward-anticipation task in the negative condition. We conducted a 2 (age: 5- or 7-years-old) × 3 (puppet: first-hand observation, positive testimony, and neutral testimony) chi-square test on the number of children in the high-reward-anticipation in the negative condition. The analysis found a significantly different pattern in the high-reward-anticipation between the two age groups, χ^2^(2) = 11.58, *p* < .01. To reveal whether the children anticipated the high reward from puppet C (in the neutral testimony video), we separately conducted a one-way likelihood ratio chi-square on the number of 5- and 7-year-olds in the high-reward-anticipation. Analysis of the 5-year-olds did not find any differences among the number of children who anticipated the high reward from the puppets in the first-hand observation video (*n* = 9), in the negative testimony video (*n* = 9), or in the neutral testimony video (*n* = 14), χ^2^(2) = 1.56, *p* = .46. On the other hand, the analysis of the 7-year-olds revealed significant differences among them, χ^2^(2) = 37.56, *p* < .001. Most children anticipated the high reward from puppet C (in the neutral testimony video; *n* = 27, *p* < .001). Only 2 anticipated the high reward from puppet A (in the first-hand observation video), and 3 anticipated it from puppet B (in the negative testimony video). Our next question concerned whether the impact of negative testimony would be higher or lower than the negative information gained by first-hand observation for those who formed their attitudes based on the content of testimony (i.e., those who anticipated the high reward from puppet C in the neutral testimony video, *n* = 27). We conducted a one-way likelihood ratio chi-square on the number of 7-year-olds who anticipated the medium reward from either the puppet in the first-hand observation or in the negative testimony video (Fig 3). We found no significant difference between the number who anticipated the medium reward from puppet B (in the negative testimony video; *n* = 15) or puppet A (in the first-hand observation video; *n* = 12), χ^2^(1) = 0.33, *p* = .56, indicating the impacts of the negative information gained from first-hand observation and testimony on children’s attitudes were not different.

Finally, we conducted a Fisher’s exact test to reveal whether the children displayed a positivity or negativity bias when they anticipated rewards based on testimony. The number of 7-year-olds whose reward-anticipation was influenced by negative testimony (84%) significantly exceeded the 7-year-olds whose attitudes were influenced by positive testimony (44%), *p* = .001. We found no significant differences between the number of 5-year-olds influenced by positive testimony (34%) and negative testimony (44%), *p* = .61.

### Explicit evaluation

Table 1 represents the rating scores in each condition. A 2 (condition: positive or negative) × 2 (age: 5- or 7-years-olds) × 3 (puppet: first-hand observation, testimony with valence, and neutral testimony) ANOVA on the children’s rating scores for each puppet (each converted from -2 to 2) revealed a significant three-way interaction, *F*(2, 248) = 6.17, *p* = .01, *η2* = .01, so we separately conducted further analysis for each valence condition.

**Table 1:** Mean rating scores for puppets.

	First-hand observation *M* (*SD*)	Testimony with valence *M* (*SD*)	Neutral testimony *M* (*SD*)
Positive condition			
5-year-olds	1.31 (0.97)	1.28 (1.05)	1.03 (1.09)
7-year-olds	1.81 (0.40)	1.59 (0.61)	0.78 (0.71)
Negative condition			
5-year-olds	-0.59 (1.36)	-0.38 (1.39)	1.22 (0.79)
7-year-olds	-1.25 (0.62)	-1.06 (0.88)	1.25 (0.80)

Testimony with valence means positive and negative testimony in each condition. Scores range from -2 (very bad) to 2 (very good).

A two-way ANOVA (age × puppet) on the positive condition found a significant main effect for puppet, *F*(2, 124) = 15.00, *p* < .001, *η*^*2*^ = .10, although the main effect for age was not significant, *F*(1, 62) = 1.54, *p* = .22, *η*^*2*^ = .01. Since the interaction between age and puppet was significant, *F*(2, 124) = 4.70, *p* = .01, *η*^*2*^ = .03, we conducted a post-analysis of the simple effects of interaction, but found no significant difference in the 5-year-olds’ rating scores across the three puppets, *F*(2, 62) = 1.03, *p* = .36, *η*^*2*^ = .01. However, the 7-year-olds’ rating scores were significantly different across them, *F*(2, 62) = 31.77, *p* < .001, *η*^*2*^ = .37. A Holm’s sequentially rejective Bonferroni post-hoc test revealed that the participants rated puppet B (in the positive testimony video) more positively than puppet C (in the neutral testimony video; *p* < .001). Their ratings for puppet A (in the first-hand observation video) were also higher than for puppet C (in the neutral testimony video; *p* < .001). The ratings for puppet B (in the positive testimony video) and puppet A (in the first-hand observation video) were not significantly different (*p* = .13). These results show that only the 7-year-olds evaluated others based on the content of positive testimony.

In the negative condition, a two-way ANOVA found a significant main effect for age, *F*(1, 62) = 10.06, *p* < .01, *η*^*2*^ = .02. The 5-year-olds’ overall ratings were somewhat higher than those of the 7-year-olds. We also found a significant main effect for the puppet, *F*(2, 124) = 82.76, *p* < .001, *η*^*2*^ = .47. A Holm’s sequentially rejective Bonferroni post-hoc test revealed that the children’s ratings of puppet C (in the neutral testimony video) were higher than puppet B (in the negative testimony video; *p* < .001) or puppet A (in the first-hand observation video; *p* < .001). The rating scores of puppet B (in the negative testimony video) and puppet A (in the first-hand observation video) were not significantly different (*p* = .33). Nor was the interaction between age and puppet significant, *F*(2, 124) = 2.41, *p* = .09, *η*^*2*^ = .01. Both the 5- and 7-year-old children evaluated others based on negative testimony.

## Discussion

In this study, we examined whether 5- and 7-year-olds’ attitudes were influenced by testimony about others and whether the valence of testimony affected their attitudes differently. We found that positive testimony only influenced the 7-year-olds’ explicit evaluations, whereas negative testimony affected the explicit evaluations (i.e., attitudes at a cognitive level) of both the 5- and 7-year-olds and the 7-year-olds’ reward-allocation and anticipation (i.e., attitudes at a behavioral level).

The impact of testimony on the children’s behavioral attitudes was evident in 7-year-olds, not in 5-year-olds. This finding is consistent with Lane et al. [15], where the children’s use of testimony to form their own attitudes toward an agent was expressed by their drawings that emerged by the age of 7. However, Haux et al. [12] concluded that even 5-year-olds are influenced by negative testimony when they choose a cooperative partner. Our result contradicts their work, probably because of an association between the testimony content and the experimental task in which the children were engaged. In Haux et al.’s study [12], the testimony content was directly associated with their experimental task. The children heard testimony that described a puppet as always uncooperative in a clown game and were then asked whether they wanted to choose the puppet as a partner for that clown game. On the other hand, we provided the children with testimony that was irrelevant to the tasks that measured their attitudes, as in Lane et al. [15]. Our null result in 5-year-olds might be explained by such methodological differences from Haux et al. [12].

Our results found no evidence that 5-year-olds form positive or negative behavioral attitudes toward others based on the content of testimony. However, they did rate the goodness of the agents when they were explicitly asked after being exposed to negative testimony; in other words, they were able to attribute moral traits to others. From this result, we conclude that they formed attitudes toward others based on negative testimony at a cognitive level. This suggests that they may possess an ability to understand and process the content of negative testimony, a result that is consistent with previous studies [14, 15], because attributing moral traits based on information obtained by testimony could reflect an ability to appropriately process verbal information [25]. This interpretation implies that children first learn to process the information gained by testimony and use it to distinguish between benevolent or malevolent persons at a cognitive level (measured by explicit evaluations in our study) at the age of 5. At the age of 7, they learn to use testimony to form their attitudes at a behavioral level (measured by reward tasks in our study), perhaps based on the prior discrimination or evaluations of others. Forming such attitudes as rewarding others or anticipating rewards from others takes more time after children have acquired the ability to process testimony content.

We also found that negative testimony has a greater impact on children’s attitudes than positive testimony, which indicates that they exhibited negativity bias. Detecting and avoiding antisocial people is crucial because interaction with such people may be threatening. Having such a disposition is beneficial not only for an individual’s welfare but also for the prosperity of social groups because detecting, punishing, ostracizing, or policing violators protects a group cooperative [26, 27]. Indeed, previous studies found that children’s attitudes toward others are more influenced by negative information. For example, their selective trust [19] and direct reciprocity [18] were affected more by negative than positive information. When children form attitudes that are relevant to their welfare or social consequences, negative testimony might be crucial.

Finally, the impact of negative testimony on 7-year-olds differed depending on whether they were allocators or receivers of rewards. When they rewarded others, they gave more valuable rewards to those whose antisocial behaviors were conveyed by testimony than those whose antisocial behavior they had witnessed themselves. On the other hand, they valued testimony to the same degree as information gained through first-hand observations when they chose a partner from whom they were more likely to confer greater rewards to themselves. These results indicate that 7-year-olds’ attitudes are more biased by negative information gained through first-hand observations rather than by testimony when they are reward-allocators. No such tendency is applicable when they receive benefits. Perhaps 7-year-olds’ sensitivity to negative verbal information depends on the kinds of attitudes they show others.

Our work does have some methodological limitations. As in previous studies [2, 12, 28], we used human-like puppets as informants and agents. However, to assure ecological validity, future research should evaluate how children react to and use testimony when both the informant and the testimony’s subject are humans. The second limitation is that we only used two kinds of positive testimony, both of which are related to helping behaviors. Although positive testimony overall has less impact on children’s attitudes, a possibility remains that positive testimony might influence children when it contains highly valent prosocial behavior, for example, devoting oneself to protecting victims against aggressors [29]. More scrutiny is needed to completely understand the development of its impact. Finally, we measured children’s relative attitudes rather than their absolute attitudes. Children more frequently face situations where they need to decide how they will behave toward one person rather than being forced to choose among options. Subsequent research would need to measure their absolute attitudes after exposure to testimony about people to assure ecological validity.

In conclusion, children’s tendency to be influenced by testimony emerges at the age of 7. Negative testimony influences their attitudes more than positive testimony. The 7-year-olds’ use of testimony differs depending whether they are the allocators or receivers of rewards. Our findings elucidate the impact of verbal information on children when they navigate their social world and offer insight into our understanding of how they interact with others using verbal information about the social agents around them without first-hand observations. These implications could also be important to identify what kinds of information the children value when they build relationships with peers. Further investigation is needed to understand the impact of testimony on children’s attitudes by elucidating situations where they need to consider the credibility as well as the intentions of informants.

## Supporting information

S1 Table Order of presentation of video content, role of each focal puppet, and video content. (DOCX)

S2 Table Raw data. (CSV)

S3 Table Data of pilot study. (CSV)
