# Supplementary material for: The impact of positive and negative testimony on children’s attitudes toward others
Source: PLoS One. 2021 Dec 22;16(12):e0261075. doi: 10.1371/journal.pone.0261075 (PMC8694454; doi:10.1371/journal.pone.0261075)
Supplement: S1 Table — (DOCX) [file pone.0261075.s001.docx]

**S1 Table. Order of presentation of video content, role of each focal puppet, and video content.**

***Positive condition***

|  | Presentation Order | | |
| --- | --- | --- | --- |
|  | 1  Condition Name (Puppet Role, Video Content) | 2  Condition Name (Puppet Role, Video Content) | 3  Condition Name (Puppet Role, Video Content) |
| A | First-hand Observation (Green, Pencil) | Positive Testimony (Red, Ball) | Neutral Testimony (Yellow, Walk) |
| B | Neutral Testimony (Red, Swing) | First-hand Observation (Yellow, Pencil) | Positive Testimony (Green, Ball) |
| C | Positive Testimony (Yellow, Ball) | Neutral Testimony (Green, Swing) | First-hand Observation (Red, Pencil) |
| D | First-hand Observation (Red, Ball) | Neutral Testimony (Green, Walk) | Positive Testimony (Yellow, Pencil) |
| E | Positive Testimony (Green, Pencil) | First-hand Observation (Yellow, Ball) | Neutral Testimony (Red, Swing) |
| F | Neutral Testimony (Yellow, Walk) | Positive Testimony (Red, Pencil) | First-hand Observation (Green, Ball) |

***Negative condition***

|  | Presentation Order | | |
| --- | --- | --- | --- |
|  | 1  Condition Name (Puppet Role, Video Content) | 2  Condition Name (Puppet Role, Video Content) | 3  Condition Name (Puppet Role, Video Content) |
| A | First-hand Observation (Green, Block) | Negative Testimony (Red, Ball) | Neutral Testimony (Yellow, Walk) |
| B | Neutral Testimony (Red, Swing) | First-hand Observation (Yellow, Block) | Negative Testimony (Green, Ball) |
| C | Negative Testimony (Yellow, Ball) | Neutral Testimony (Green, Swing) | First-hand Observation (Red, Block) |
| D | First-hand Observation (Red, Ball) | Neutral Testimony (Green, Walk) | Negative Testimony (Yellow, Block) |
| E | Negative Testimony (Green, Block) | First-hand Observation (Yellow, Ball) | Neutral Testimony (Red, Swing) |
| F | Neutral Testimony (Yellow, Walk) | Negative Testimony (Red, Block) | First-hand Observation (Green, Ball) |
